# Tree growth potential and its relationship with soil moisture conditions across a heterogeneous boreal forest landscape

**DOI:** 10.1038/s41598-024-61098-z

**Published:** 2024-05-09

**Authors:** Johannes Larson, Carl Vigren, Jörgen Wallerman, Anneli M. Ågren, Alex Appiah Mensah, Hjalmar Laudon

**Affiliations:** 1 Department of Forest Ecology and Management, Swedish University of Agricultural Sciences, Skogsmarksgränd 17, 901 83 Umeå, Sweden; 2 Department of Forest Resource Management, Swedish University of Agricultural Sciences, Skogsmarksgränd 17, 901 83 Umeå, Sweden

**Keywords:** Forestry, Forestry

## Abstract

Forest growth varies across landscapes due to the intricate relationships between various environmental drivers and forest management. In this study, we analysed the variation of tree growth potential across a landscape scale and its relation to soil moisture. We hypothesised that soil moisture conditions drive landscape-level variation in site quality and that intermediate soil moisture conditions demonstrate the highest potential forest production. We used an age-independent difference model to estimate site quality in terms of maximum achievable tree height by measuring the relative change in Lorey’s mean height for a five year period across 337 plots within a 68 km^2^ boreal landscape. We achieved wall-to-wall estimates of site quality by extrapolating the modelled relationship using repeated airborne laser scanning data collected in connection to the field surveys. We found a clear decrease in site quality under the highest soil moisture conditions. However, intermediate soil moisture conditions did not demonstrate clear site quality differences; this is most likely a result of the nature of the modelled soil moisture conditions and limitations connected to the site quality estimation. There was considerable unexplained variation in the modelled site quality both on the plot and landscape levels. We successfully demonstrated that there is a significant relationship between soil moisture conditions and site quality despite limitations associated with a short study period in a low productive region and the precision of airborne laser scanning measurements of mean height.

## Introduction

Forest growth rate is a key aspect of forested ecosystems, and is influenced, among other things, by the complex and dynamic interactions among environmental factors that vary depending on local biotic and abiotic conditions. On both global and regional scales, climate and soil conditions represent some of the most influential factors that explain spatial variation in forest growth. Forest management adds further complexity to landscape variation of forest properties by altering important forest characteristics such as age, structure, and species distribution^1^. It is important to note that both unmanaged and managed forest areas are also affected by natural disturbances such as forest fires, windstorms, and insect outbreaks. As such, untangling the complex interactions between the environmental drivers that regulate forest growth constitutes a grand scientific challenge. This is particularly relevant for the managed boreal forests of northern Europe, where the expansive forested landscape has been managed for several hundred years; this has resulted in a patchwork of human-induced actions and natural disturbance that exert significant influences on the regulation of growth rate.

Within boreal landscapes, large variations in forest growth and carbon sequestration have been observed across short distances^2,3^. Furthermore, previous studies have identified topographic position as a key factor for the variation in soil moisture conditions, which regulate soil development, nutrient accumulation, and vegetation patterns^4–7^. Therefore, on the local landscape scale—where climate drivers such as temperature and precipitation can be considered constant—the topographic position at a specific location may largely affect the forest growth potential as a result of the differences in accumulated water surrounding areas^8^.

Site quality is the combination of the physical and biological factors of a geographical location or site. Site quality is inherent to the site, but may be influenced by management or e.g. climate change^9^. Site quality can be used to describe tree growth potential at a specific site. The fraction of a site’s growth potential that is realised by trees to produce a certain amount of volume is often quantitatively expressed as site productivity. This type of information is critical for forest management planning as it provides the context for projecting forest production over a certain period^10^ and supports decision-making concerning both conservation and restoration efforts^11^. As such, landscape-scale information on the variation in forest site productivity can significantly improve forest management^12^ and enhance understanding of which biotic factors influence forest growth within the landscape.

In even-age stands, the relationship between tree height and age of a given species is closely related to the capacity of the site to produce woody biomass^13^. Therefore, site index, which is defined as the expected height of the dominant trees at a reference age is a commonly used indicator for site productivity. In Sweden, site index is generally estimated using two main methods: (1) by height development curves and (2) by site factors. Method 1 uses the height and age of the dominant trees (i.e., the 100 largest trees in diameter per hectare) to estimate the expected height at a reference age (e.g. 100 years for Scots pine—*Pinus sylvestris* L*.* and Norway spruce—*Picea abies* (L.) H. Karst, and 50 years for birch—*Betula pendula* Roth. and *Betula pubescens* Ehrh*.*)^10,14,15^. This method of estimating site index is denoted as SIH. The requirement of information about dominant height and age means that the method cannot be applied to all forest lands in Sweden (e.g., after clear-cut or thinning from above). Therefore site index is often assessed using method 2, which is based on a combination of site factors including climate, field vegetation, location and soil properties^16^. This method of estimating site index is denoted as SIS. SIS, although age-independent, is in comparison to SIH found to be lower in accuracy (~ 4 m). Using the abovementioned methods for landscape-scale assessment of site quality poses several challenges because the methods require homogeneous stand conditions (i.e., the methods are age-and/or species-dependent), commonly not available on the landscape scale. Furthermore, a major limitation of both methods is that they are limited to fixed sample plots or field registers, which effectively constrains the potential landscape-wide extrapolation. Hence, an approach that is age-and species-independent has the potential to provide unbiased assessments of the variation in site productivity across a broader scale^17^.

When two measurements in time are available, age-independent difference equations have satisfactorily been used to model site productivity. For example, Tomé et al.^17^ developed age-independent difference equations for both dominant height (*Eucalyptus globulus* Labill*.*) and DBH (*Quercus suber* L.) growth in Portugal by reformulating well-established theoretical growth functions. This approach provides a possibility for assessing the variation in forest growth potential when age is unknown, in some cases even with higher accuracy in comparison to age-dependent methods^18^. Furthermore, this approach facilitates landscape scale assessment of the variation in forest productivity using remote sensing when two measurements in time are available.

Remote sensing, particularly airborne laser scanning (ALS), has rapidly advanced during the last decade. The use of ALS in resolving the three-dimensional properties of forest vegetation structure has shown great potential for measuring and estimating key attributes, such as forest growth and site productivity, at the landscape scale^19–22^. Furthermore, bi-temporal ALS data can be highly beneficial as this information can be used to reduce uncertainties related to disturbance from management (e.g., thinning, clear-cutting, etc.) and facilitates the precise estimation of site productivity through the added information of growth between periods^23^. In parallel with the developments of high resolution remote sensing for measuring forest attributes, ALS data has massively increased the resolution of topographical information and has become an essential tool for modelling soil moisture conditions on a landscape scale^24^. Landscape scale information of environmental factors such as soil moisture, provides large opportunities to study its effect on site quality. For example, Mohamedou et al.^25^ demonstrated how modelling soil moisture conditions based on terrain indices can increase the accuracy of site productivity estimates in boreal forests.

At present, landscape assessments of the variation in site quality and its relation to environmental drivers are rare, in particular across small landscapes. Within smaller spatial scales, certain environmental factors, and the interactions among them, remain constant, allowing researchers to concentrate on a specific subset of environmental drivers. Studying how variation in soil moisture conditions influences site quality may provide important insights into how environmental drivers affect forest growth, as well as enhance our ability to predict where water availability will limit tree growth potential. This type of knowledge is highly relevant for the scaling of forest ecosystem processes and development of sustainable forest management approaches in the future. Unfortunately, datasets appropriate for site quality estimation across smaller landscapes are rare. In the present study, we bridge this gap by using high-resolution, bi-temporal forest growth data to assess site quality on a landscape scale, information which is then used to investigate how site quality is related to topography-derived soil moisture conditions.

The presented research was conducted to test the following hypotheses: (1) spatial variation in soil moisture drives landscape-level variation in site quality; and (2) areas with intermediate soil moisture conditions demonstrate the highest potential forest production. To test these hypotheses, we first developed an age-independent estimate of site quality based on repeated forest surveys (2014–2019) with a 5 year study period. In the second step, site quality was estimated by using bi-temporal ALS data from the previously fitted site quality model. Thereafter, site quality was evaluated on plot and landscape level under differing soil moisture conditions. Finally, we discuss how the obtained results provide evidence for the connection between soil moisture conditions and forest production in a managed, heterogeneous boreal landscape.

## Methods

The study approach was generally centred on analysing the variation in site quality using soil moisture conditions. The estimation of site quality was based on the principle of age-independent difference equations using two measurements in time of Lorey’s mean height. The approach for site quality estimation was carried out in three main steps: (1) global parameters were estimated using a difference equation adjusted for relative height from field measurements. In the second step (2) we reformulated the fitted equation to estimate plot specific site quality estimates. In step (3), landscape estimation of site quality was made using the model from step (2) and repeated measurements from ALS as input data. Finally, the variation in estimated site quality for both on a plot and landscape scale was analysed in context of soil moisture conditions obtained from field survey and map predictions.

### Site description

The study was carried out in the Krycklan Catchment Study^26^ which is located in northern Sweden (64° 14′ N, 19° 46′ E) which covers a 68 km^2^. The area consists of a managed forest landscape with a mosaic of wetlands and lakes, typical for the region. The mean annual temperature of the area is 2.4 °C, with a mean annual precipitation of 636 mm year^−1^ based on 30 years of data (1991–2021). The catchment has a gently undulating terrain, with elevations ranging from 127 to 372 m above sea level. The upper parts of the catchment are dominated by unsorted sediments, while glaciofluvial sorted sediments are common in the lower parts. The forest soils are predominantly iron podzols. Forest cover 87% of the area and is dominated by Scots pine (*Pinus sylvestris*) (63%) and Norway spruce (*Picea abies*) (26%), with scattered occurrence of deciduous species consisting mainly of birch (*Betula pendula* and *Betula pubescens*). Since 1922, approximately 25% of the catchment has been set aside for forest research and 1% is protected as nature reserves. Ownership of the remaining area is divided among forest companies and private owners. Forests in non-protected areas are managed by conventional rotation forestry and are predominantly even-aged, artificially regenerated, and thinned. Therefore, the area has evolved into a mosaic of stands of different ages, basal area and stocks (Table 1). The field layer vegetation is dominated by ericaceous shrubs (*Vaccinium *spp*.*) such as bilberry and lingonberry on moss mats of splendid feather moss (*Hylocomium splendens*) and red-stemmed feather moss (*Pleurozium schreberi*).

**Table 1:** Descriptive statistics of plot-level data from the Krycklan forest survey 2014 (*n* = 484).

	Lorey’s mean height (m)	Stand age^1^	Basal area (m^2^)	Volume (m^3^ ha^−1^)
Min	0	0	0	0
Median	13	64	19	129
Mean	12	69	18	139
Max	26	200	63	721

^1^Stand age (i.e., number of years since stand establishment) was determined for each sample plot as the basal area-weighted mean age obtained by coring 8–10 dominant trees outside each sample plot.

### Field data

**Figure 1:**
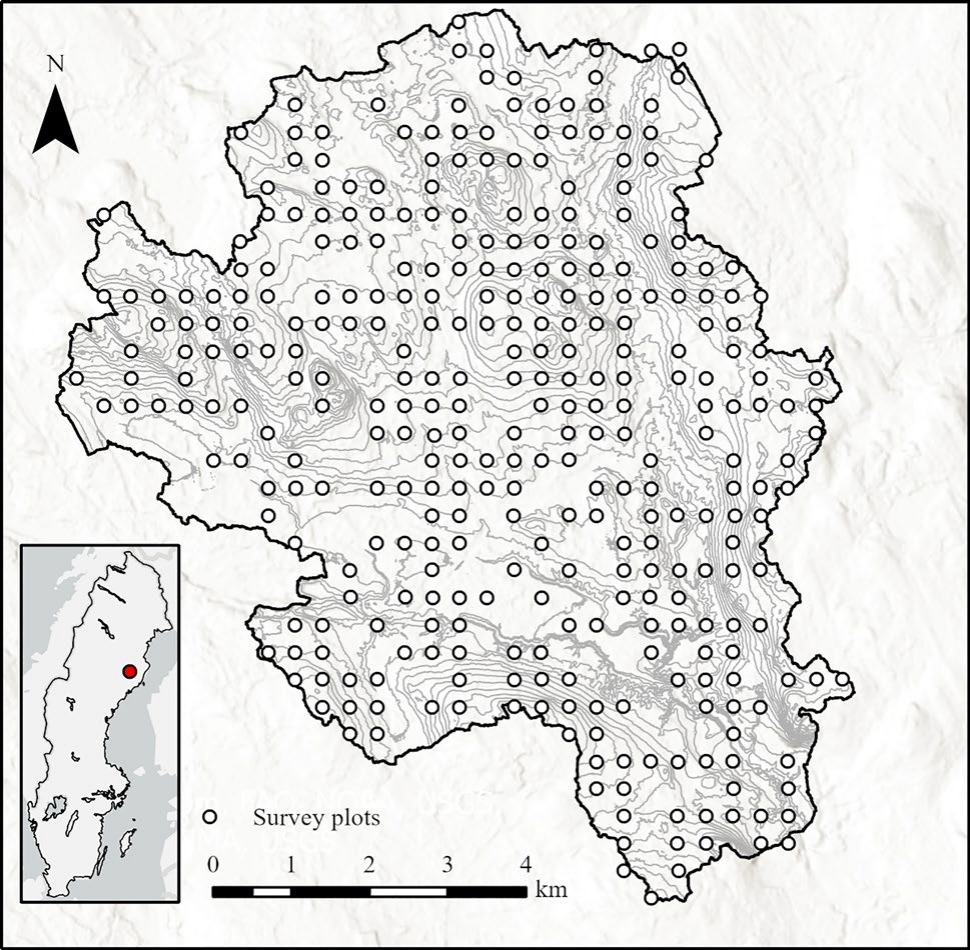
Map of the Krycklan catchment and the location of the 337 survey plots (350 × 350 m square grid). The map was created using ArcGIS Pro (version 3.0.2).

In 2014, a survey grid covering the entire catchment area was established; this grid comprises of > 500 plots (radius: 10 m, area: 314.5 m^2^) that are spaced 350 × 350 m apart (Fig. 1). The plot locations were allocated using a randomly chosen origin, which was oriented along the coordinate axis of the SWEREF 99 TM projection. Accurate centre positions for each plot were determined using differentially-corrected GPS measurements, which were obtained with a Trimble GeoXTR receiver and the SWEPOS real-time differential correction service. A forest survey was conducted in the late fall and early spring of the 2014 and 2019 growing seasons. All trees with a diameter at breast height (DBH, 1.3 m) greater than 4 cm were measured at each plot. To reduce the labour necessary for surveying, the plot radius was reduced from 10 to 5 m for stands with a high stem density (e.g., regenerating or young forests). For all measured trees, DBH, species, and tree status (live or dead) was recorded. Tree height was measured for subjectively selected undamaged sub-samples of at least three trees of each species with a laser-guided hypsometer selected to capture tree size variation in DBH for each species. The selection of sample trees was made independently at each survey occasion. We fitted a mixed-effects model with plot-level random effects, where the dependent variable was the height of the measured trees and the independent variable was their DBH. We then estimated the height of the remaining trees using these models, followed by calculating the plot Lorey’s mean height (basal area weighted mean height). Plots without measured trees such as clear-cut areas, treeless mires were removed. In addition plots with a decrease in Lorey’s mean height between the two observations were excluded, which could be caused by management, natural disturbance or the independent selection of sample trees at each survey occasion . After this exclusion, our survey data encompassed a total of 337 survey plots, each with consecutive measurements of Lorey’s mean height. In addition to the tree measurements, the plots were classified into soil moisture classes (dry, mesic, mesic-moist, moist, and wet) based on an estimation of each plot’s average depth to groundwater level during the vegetation period; these estimates were based on the position of each plot in the landscape and vegetation patterns as per protocols of the Swedish National Forest Inventory^27^. The soil types for 315 of the survey plots were determined in a soil survey completed between 2019 and 2020^28^ according to World Reference Base for Soil Resources (WRB) guidelines.

### ALS data

Airborne Laser scanning (ALS) covering the entire study area was performed adjacent to both forest survey campaigns (Table 2.). In August 2015, the study area was scanned using an Optech Titan X sensor (flight height: 1000 m) to yield an average point density of 20 points per m^2^. The sensor scanned the area using three specific wavelengths, e.g., 532 nm (green), 1064 (NIR), and 1550 nm (SWIR). At the end of June 2019, the area was scanned using a Riegl VQ-1560i-DW sensor at wavelengths of 532 nm (green) and 1064 (NIR); this yielded an average point density of 20 points per m^2^^26^.

**Table 2:** ALS data specifications.

	2015	2019
Time period	2015-08-23	2019-06-27
Season	Leaf on	Leaf on
Instrument	Optech Titan X	Riegl VQ-1560i-DW
Flying height	1000 m	1000 m
Measured wavelengths	532 nm (green), 1064 (NIR), and 1550 nm(SWIR)	532 nm (green), 1064 (NIR)
Point density	20 points/m^2^	20 points/m^2^

The raw ALS point clouds were then processed by classifying point returns as ground, vegetation, unclassified, and noise. This enabled the generation of a Digital Elevation Model (DEM) to which all of the ALS points were normalised. The point returns were aggregated to 10 × 10 m metrics using CloudMetrics Fusion software^29^. Outlier assessments, carried out using bivariate scatterplots, were performed to examine the relationship between field measurements of Lorey’s mean height and the 95th percentile height of laser returns (P95) from two scannings. Observations with a height difference > 5 m between the field measured Lorey’s mean height and P95 were excluded because these observations were considered to represent instances in which silvicultural practices, such as thinning or clearcutting had been performed between the scanning and field measurements. The number of plots excluded due to this discrepancy was 38 in 2014 and 25 plots in 2019.

**Figure 2:**
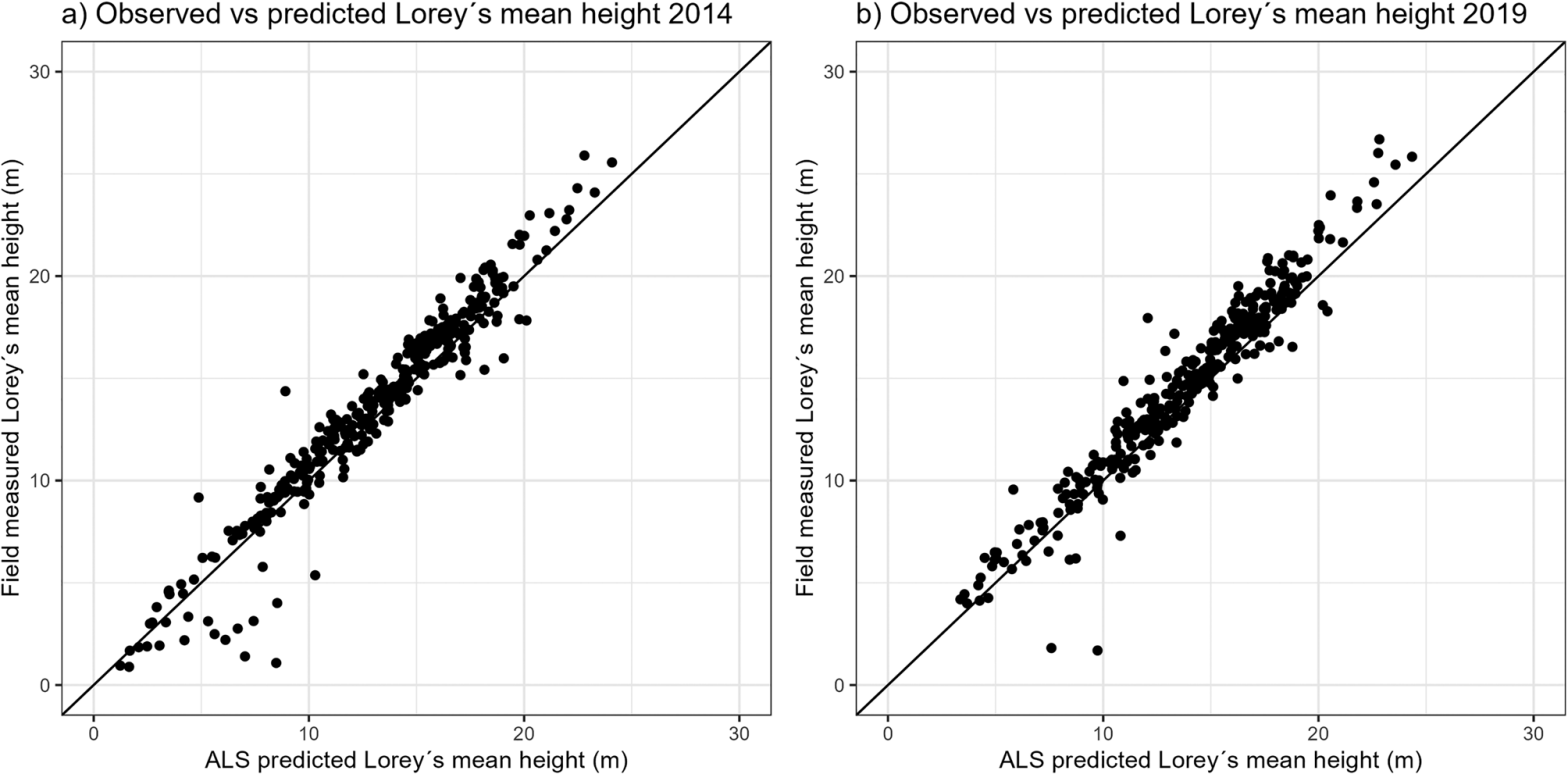
Plots illustrating the observed vs. predicted Lorey’s mean tree heights from forest surveys conducted in 2014 (**a**) and 2019 (**b**).

An area-based approach was used to obtain wall-to-wall coverage of Lorey’s mean height across the entire study area^30^. In the first step, the observed Lorey’s mean height from the geo-referenced survey plots at each survey occasion was regressed on the ALS metrics from the corresponding ALS scanning. In the second step, the models were applied over tessellations of individual grid cells to generate wall-to-wall estimates of Lorey’s mean height at time of each survey occasion (2014 and 2019). We tested different predictive models using various combinations of commonly used ALS metrics related to height and density^23,31^. The final predictive model chosen for each year was formulated as a linear regression with the same independent variables which included P95 and the standard derivation of height (heightStdDev). Both models showed high accuracy, with the residual standard error (RSE) falling below 1.1 m for both the 2014 and 2019 (Table 3). The estimations of Lorey’s mean height predicted from the individual models at each survey year, corresponded well to the field measurements (*n* = 337) (Fig. 2).

**Table 3:** Results from linear regression predicting Lorey’s mean height for 2014 and 2019 using the area-based method.

Year	Model	Adj *R*^2^	RSE (m)
2014	Height = β_0_ + β_1_(P95) + β_2_(heightStdDev) + β_3_(P95 * heightStdDev)	0.96	1.05
2019	Height = β_0_ + β_1_(P95) + β_2_(heightStdDev) + β_3_(P95*heightStdDev)	0.95	1.03

### Site quality estimate

This study required an age-independent estimation of site quality to avoid the limitations of the commonly used ‘site index’, which requires inputs such as the ages and heights of dominant trees or, alternatively, vegetation type and site properties. A mean height growth model with a sigmoidal shape will involve an asymptote and a shape parameter describing how the asymptote is reached; as such, an estimator of site quality can be deduced by expressing one of the parameters as a function of an un-observed site quality. The Richard’s growth model is suitable for this purpose as it was empirically derived from tree physiology, has desirable properties, and has been widely used in forest growth analyses^32,33^. We focused on the age-independent difference formulation (Eq. 1) of the Richard’s model^34^ presented by Tomé et al.^17^ to derive the site quality estimate:

1 $${Y}_{i+a}=A{\left\{1-{e}^{-ka}\left[1-{\left(\frac{{Y}_{i}}{A}\right)}^{m}\right]\right\}}^\frac{1}{m}$$

where *Y* is Lorey’s mean height in metres, *A* is the asymptote/maximum height (m) when time/age approaches infinity, *k* is a parameter related to the growth rate, *m* is a shape parameter related to the point of inflection, and *a* is the number of periods. The generalised model did not consider tree species. This age-independent difference formula can be used to model height growth for the estimation of site quality when two successive measurements are available; this includes the assumption that the growth function passes through the two height measurements in both survey periods. In Eq. 1, the parameter *A* is most often the parameter that is most strongly related to site quality^23^, as well as easy to interpret because it is expressed in the same dimension as the response variable, height. The *k* parameter can also be expressed as a measure of relative height growth from the field mean height measurements at time *i* and *i* + *a*, and a global parameter *b*:

2 $$k=b*\left(\frac{{Y}_{i+a}}{{Y}_{i}}\right)$$

Equation (2) was substituted into Eq. (1) to obtain estimates of the global parameters *m* and *b*. The parameters were estimated using generalised nonlinear least squares in the R Environment^35^. To derive plot-specific site quality estimates (*A*_*o*_), Eqs. (1) and (2) were algebraically reformulated (Eq. 3) as a function of the height measurements at times 1 and 2 (corresponding to the survey periods 2015 and 2019, respectively) and the global parameters *m* and *b* as:

3 $${A}_{o}={\left(\frac{{{Y}_{i+a}}^{m}-{e}^{-ka}{{Y}_{i}}^{m}}{1-{e}^{-ka}}\right)}^\frac{1}{m}$$

### Landscape site quality estimate

We used Eq. (3) to estimate the site quality parameter (*A*_*o*_) to describe the expected maximum height over the entire study area based on the ALS data, more specifically, Lorey’s mean heights from the bi-temporal ALS data. We masked roads, railroads, and powerlines to reduce noise. In addition, we masked clear-cuts from 2000 to 2020 based on data from the Swedish Forest Agency.

### Auxiliary data

To investigate variation in site quality within the study area, environmental variables describing the site properties were obtained for the 337 plots and on the landscape level. Soil moisture conditions were extracted from the continuous SLU (Swedish University of Agricultural Science) soil moisture map that describes variation in soil moisture conditions across Sweden^24^. The map was produced using machine learning, more specifically, by combining geographically mapped information, e.g., various ALS-derived terrain indices, climate data, and quaternary deposit information. The most important predictors of the developed soil moisture model was the Depth to water index (DTW), the Topographic wetness index (TWI), and mapped information of wetlands. The training and validation data sets included almost 20,000 survey plots with soil moisture classifications across Sweden, and the final mapped information expresses the probability that a 2 × 2 m pixel is classified as wet (0–100%). Ågren et al.^24^ previously used the survey plots included in this study as an independent validation dataset. For the present study, the 2 × 2 m resolution available in the SLU map was resampled to a 10 × 10 m grid using bilinear interpolation to match both the resolution of the field plots and the ALS metrics over the study area.

### Statistical analyses

After assessing the spatial autocorrelation of plots using a semivariogram (Fig. S1), we concluded that each plot could be considered as an independent observation. To address the first hypothesis, i.e., that soil moisture drives variation in site quality at the landscape scale, we used second-order polynomial regression to examine the relationship between site quality and modelled soil moisture. To address the second hypothesis, we performed a non-parametric Kruskal–Wallis test^36^, followed by a Dunn-Bonferoni test^37^, to test for significant differences in estimated site quality between pairs with different soil moisture classifications, soil types and dominating species. The Kruskal–Wallis test was chosen because not all of the groups fulfilled the assumption of a normal distribution and the presence of differences in sample sizes^38^. All of the statistical analyses were conducted using R software^35^.

## Results

**Figure 3:**
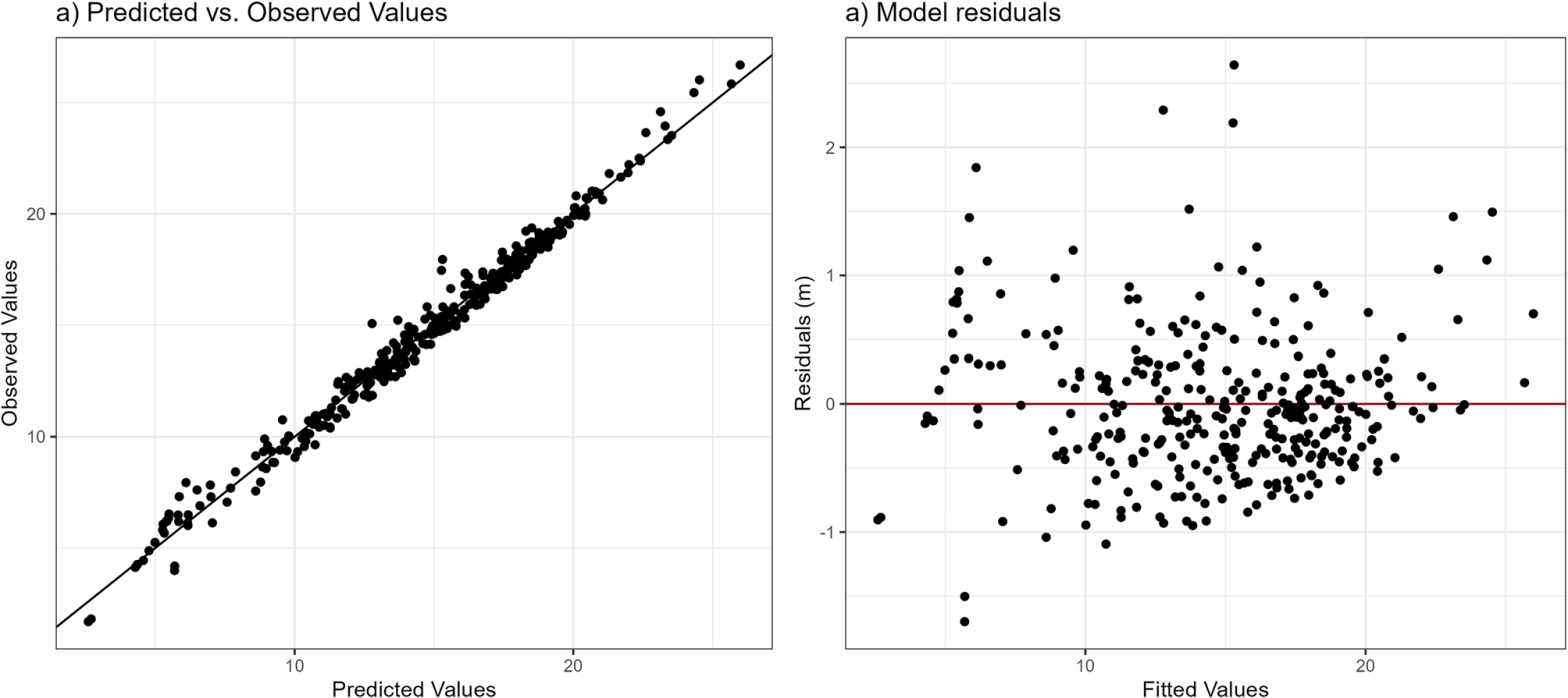
Plot of the predicted vs. observed Lorey’s mean height values (**a**), and residuals from the predicted model in comparison to observed values (red line) (**b**).

The growth in tree height between the two surveys for individual survey plots ranged from 0 to 4.8 m, with a mean of 0.8 m; the median relative height growth was 6%. We fitted the age-independent difference equation (Eq. 1) on the complete dataset (*n* = 337 field plots) using generalised nonlinear least squares to obtain estimates of the global parameters, denoted as *m, b* and *A*_global_ (Table 4). The overall model was significant and showed a good fit, with a residual standard error of 0.41 m. In addition, the model errors did not show any obvious signs of heteroscedasticity (Fig. 3).

**Table 4:** Estimated parameters of model 1, based on the field data.

Parameter	Estimate	Std. error	*t*-value	Fit statistics			
				AIC	BIC	logLink	RMSE(m)
*A*_*global*_	26.99	1.70	15.88	576.85	592.13	− 284.43	0.41
*b*	0.021	0.00	6.24				
*m*	0.57	0.10	5.53

**Figure 4:**
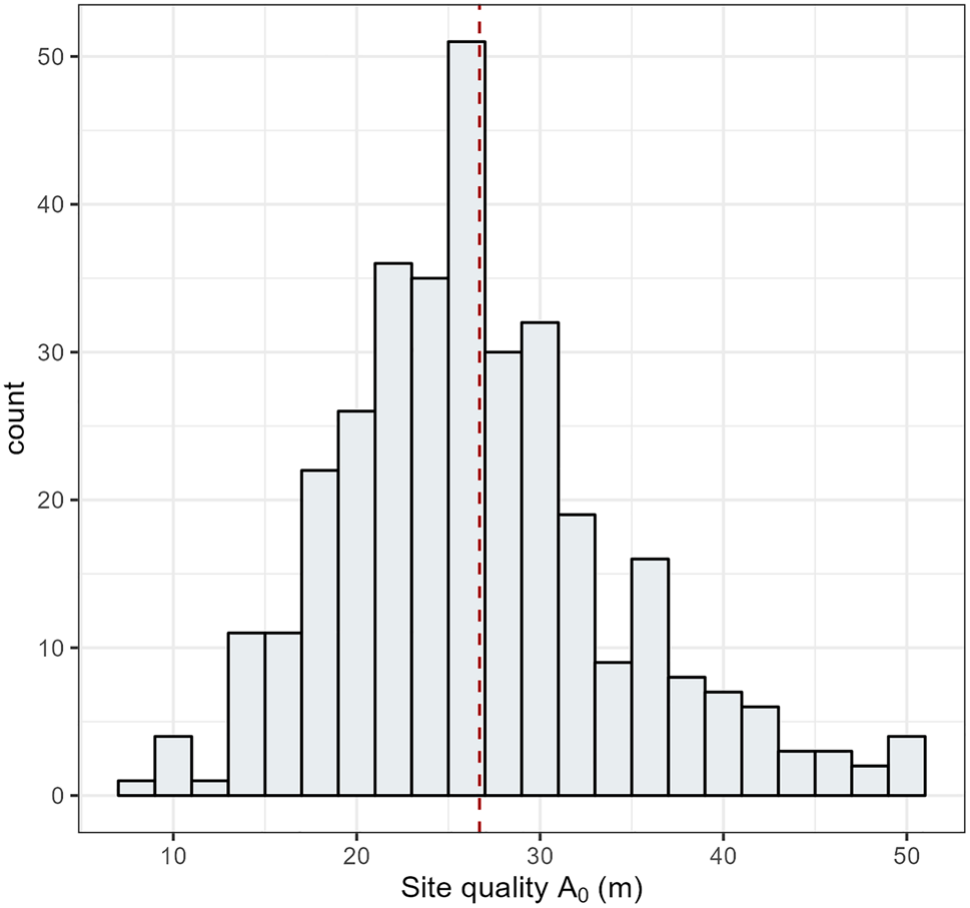
Histogram of estimated site quality (*A*_0_). Note: all observations over 50 m were placed in the 48–50 m class.

The estimated global parameters *m* and *b* were used to estimate site quality (*A*_*o*_, or the expected maximum height) for each survey plot using the algebraic solution for *A*_*o*_ (Eq. 3). The estimated site quality (*A*_*o*_) had a mean of 25.9 m and ranged from 7.2 to 67.3 m (Fig. 4).

**Figure 5:**
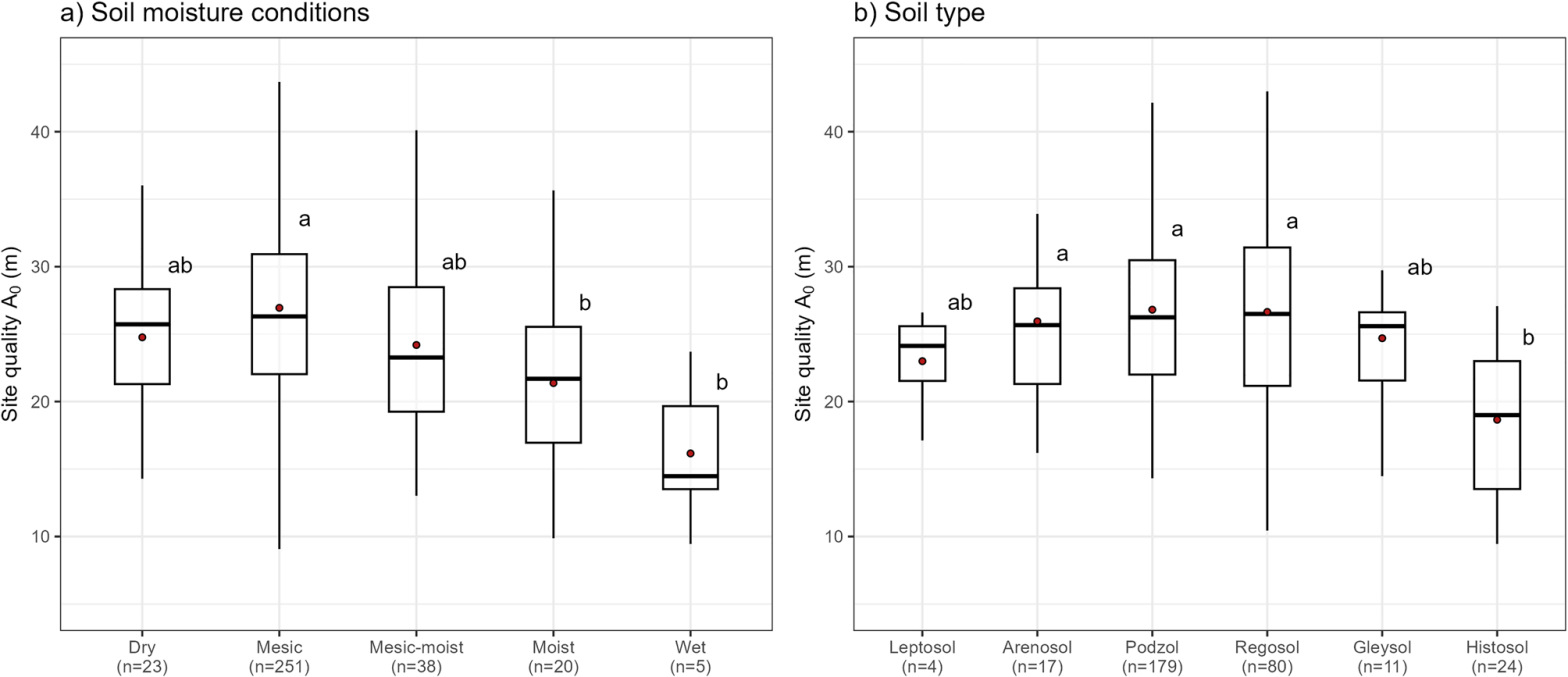
The relationship between site quality and (**a**) soil moisture conditions and (**b**) soil type. Lowercase letters show the results from the corresponding Dunn-Bonferoni test.

We found significant differences in site quality among plots with different classified soil moisture conditions (Kruskal–Wallis chi-squared = 24.633, df = 4, *p*-value < 0.001), with the highest potential forest production found in areas with intermediate soil moisture conditions (Fig. 5a). Moreover, mesic sites showed significantly higher site quality in comparison to moist and wet soil moisture classes. Significant differences in site quality were also observed for plots characterised by different soil types (Kruskal–Wallis chi-squared = 29.464, df = 5, *p*-value < 0.001), with histosols showing significantly lower site quality values in comparison to arenosols, podzols, and regosols in the post-hoc Dunn-Bonferoni test (Fig. 5b).

**Figure 6:**
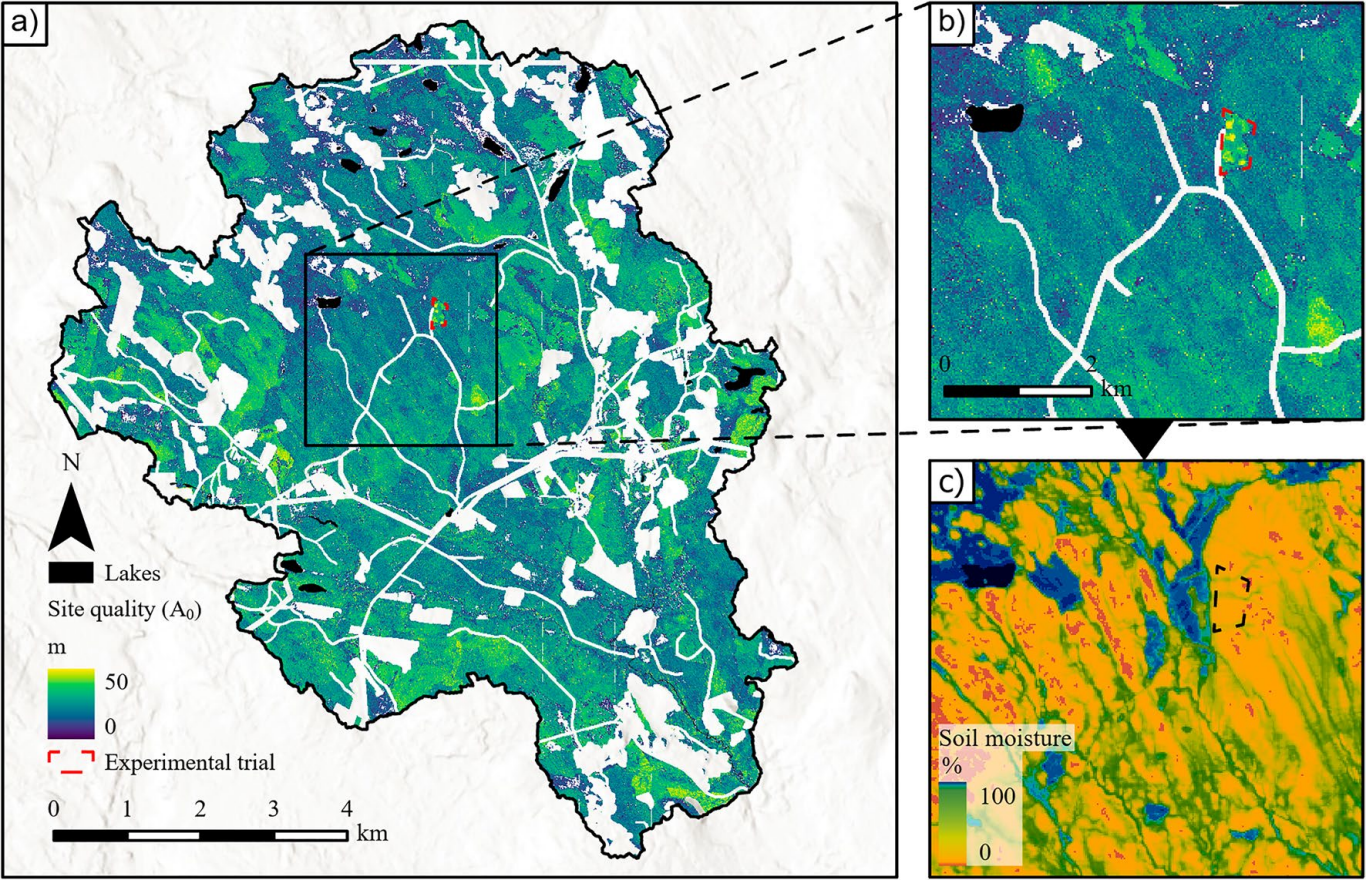
Predicted site quality across the Krycklan catchment, based on bi-temporal ALS data (**a**). Predictions of site quality in a smaller area (**b**), with the SLU soil moisture map over the same area (**c**). White areas are masked areas such as clear-cuts, roads. agricultural fields, or and power lines. An experimental trials of different tree species bordered with red (black in c). The map was created using ArcGIS Pro (version 3.0.2), https://www.esri.com/en-us/arcgis/products/arcgis-pro/overview.

Equation (3), when applied to the landscape level, used bi-temporal ALS estimates of mean height to compute the expected site quality (i.e., maximum height) for each pixel (10 × 10 m spatial resolution). Estimated site quality using bi-temporal ALS data demonstrated a near-normal distribution that included a similar range as the estimated site quality using field data. The visual comparison of modelled soil moisture and site quality revealed noticeable patterns (Fig. 6). For example, areas with low site quality estimates generally showed rather wet soil moisture conditions, and thus, were located in areas dominated by peat soils. On the other hand, some areas showed higher site quality in comparison to neighbouring areas, in some cases likely associated with different dominating species. For example, clear differences in site quality could be observed within an experimental trial with blocks of different tree species (Fig. 6b). Stand edges could also be observed where younger stands showed higher site quality in comparison to mature stands. An effect of tree species was in line with the observations from field data plot scale, where plots dominated by *Pinus contorta* showed a significantly higher mean site quality than plots dominated by other tree species (Fig. S2).

**Figure 7:**
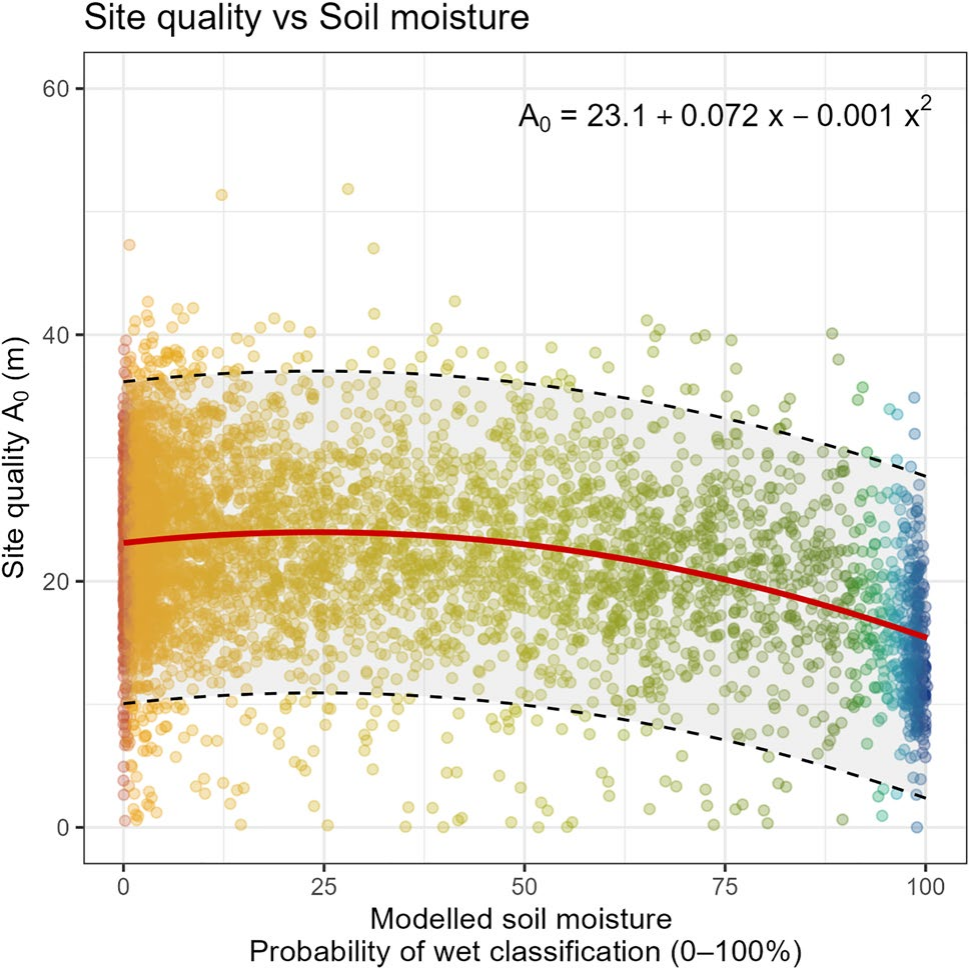
The modelled polynomial relationship between site quality (*A*_0_) and soil moisture across the entire study area. The plot displays a random sample of 5000 (10%) raster cells, coloured corresponding to the SLU soil moisture map (Fig. 6c). The regression line is shown in red, with the dashed lines representing the 95% prediction intervals. The modelled soil moisture, shown as a percentage, denotes the probability of the point being predicted as wet rather than the volumetric soil water content.

At the landscape scale, the relationship between site quality and the modelled soil moisture conditions (the probability of a point being classified as wet) was described by a second-degree polynomial regression model (*R*^2^ = 0.11, *p*-value < 0.001, F-stat = 31,380); indicating that site quality decreases as soil moisture increases. However, the estimated site quality showed large variation in relation to the predicted soil moisture condition (Fig. 7).

## Discussion

Understanding the factors that explain variations in site quality across a certain landscape is a tremendous scientific challenge due to the complex interactions between different environmental drivers varying in importance across scales. This study focused on a 68 km^2^ meso-scale heterogeneous landscape, a decision which effectively controlled for dominant environmental drivers that are present on the national and regional levels, including climatic gradients. We estimated forest site quality across the Krycklan catchment by using an age-independent difference approach based on repeated and extensive field measurements of mean height. To estimate site quality over the entire landscape, we applied our age-independent model to the available bi-temporal ALS data, which had been obtained in close succession to the field plot surveys. The landscape estimates of site quality were compared to readily available auxiliary data to assess the effects of soil moisture conditions on forest production potential in the study area.

The use of bi-temporal ALS data to estimate forest growth and site quality has gained momentum in recent years, and has already proven successful in multiple previous studies using varying approaches^19,39,40^. Previous researchers have emphasised that age-independent models can rely on ALS data to yield landscape assessments of site quality across various time periods without the constraint of age-specific information for trees^22,23,39^. For instance, landscape estimates of site productivity, commonly presented using the site index, become challenging when information on forest age, species distribution, or top/dominant height or site properties is limited to field plots. Furthermore, the site index can only be directly estimated if the stand meets standard assumptions such as level of stocking, species composition, and that the stand history has not excessively affected the development of the dominant/co-dominant tree species.

The methodological differences between the present study and previous research mean that direct comparisons of our results to what has been reported in prior years are challenging. However, it is possible to compare the results obtained from our age-independent model for tree height, which was used to estimate the global parameters and derive the site-independent site quality estimates. The age independent height model predicting height at time two, showed satisfactory accuracy with a RMSE of 0.41 m (Table 4). This is in line with the model performance (RMSE value of 0.64 m) reported for an age-dependent Chapman-Richard function with RMSE of 0.64 m^41^.

The site quality estimates reported in this study represent the site-specific maximum attainable Lorey’s mean height when time/age approaches infinity. This theoretical value cannot be validated within the boundaries of this study and is limited to be used to compare differences in site quality between sites. At the same time, the site quality estimates showed a large range from 7 to > 50 m, where especially the values in the highest can be considered to be unreasonable. Furthermore, the current study was limited to information from only two field survey inventories that were separated by a period of 5 years. This can be considered as a short growth period, and the two measurements may not include all of the information necessary to explain the variation in site quality, especially if the measurements do not contain the empirical asymptote. The study period in the presented research is far from the period needed to begin reaching the asymptote, that is, the maximum value of the site quality parameter. It is also important to note that the use of tree height (ascertained through the height-age site index approach) does not fully explain between-site differences in productivity. In other words, even if we have a certain site index, there may be significant variation in the woody volume of the plot due to differences in carrying capacity, species composition, and site properties^10,13,42,43^. Thus, we postulate that additional information to height differences, such as species, basal area, or volume, may improve both site index or site quality estimates^13^. However, such information—especially volume—is not as readily available as tree height, which is available for the whole of Sweden due to enhanced forest inventories. Another advantage of using height is that this metric, in comparison to basal area and volume, is not heavily affected by management and/or stand density. Nevertheless, a previous study based on bi-temporal ALS data reported stand density effects on *P. sylvestris* height growth in highly productive stands^44^.

After the site quality model had been fitted to the data set describing the survey plots (Eq. 3), it was then applied to the bi-temporal ALS data to compute the expectedsite quality for 10 × 10 m raster cells of the study area. The different laser scanners used in the 2015 and 2019 surveys could have caused potential errors in the pixel-level estimations of site quality (Table 2). However, we would like to argue that this potential source of error is small due the two separate calibration for each scanning based on field measured Lorey’s mean height. Other potential sources of errors in the site quality estimates include the unavailability of tree species information at the landscape level, along with the effects of forest management. The magnitude of the effects of these error sources warrants further investigation. Another source of uncertainty is associated to a short study period in combination with the low productive forests in the region, which may not have been sufficient to capture the variation in site quality. The average field measured Lorey’s mean height difference was 0.8 m in the studied period. Ground-based tree-height measurements of pine and spruce involve a mean error of 0.3 and 0.1 m, respectively^45^, which may limit the observation of between-site differences. Gathering landscape-level species information has the potential to improve site quality estimates, which was apparent in the higher estimates of site quality in an experimental tree species trial (Fig. 6a). Furthermore, the observed stand edges with higher site quality associated to younger stands may be an effect of a higher proportion of birch, which is a fast growing pioneer species^46^. Previous studies have found that dividing the study area into strata based on tree species improves prediction accuracy^47^.

The reported results were consistent with our hypothesis in that site quality decrease in high soil moisture conditions. Histosols in this region are associated with saturated soil conditions and showed significantly lower site quality in comparison to other soil types. Similar findings for soil moisture conditions and soil types have been observed in studies of the relationship between normalised mean annual increment in relation to soil properties across Sweden^48^. Previous studies have also proven the utility of soil moisture maps in comparison to other terrain indices^49^, as well as the ability to predicting thick organic soil layers based on soil moisture^50^. Lower site quality is expected in these areas because the saturated soil conditions decrease tree growth^48,51^. Notably, the modelled soil moisture was not able to explain the variation in soil quality among drier areas. We suspect several reasons for this. Firstly, the soil moisture map used in this study was create to differentiate between the soil moisture classes, e.g., dry (dry and mesic) and wet (mesic-moist, moist and wet); this may not adequately capture the variation within these groups observed on plot level (Fig. 5)^24^. The use of remote sensing technologies in combination with additional auxiliary data is not a new phenomenon. When site index was modelled for *Pinus pinaster* Ait. stands in Spain, climate-related factors such as potential evapotranspiration, mean minimum temperature, and mean precipitation were among the most important variables that explained variation in a site-specific quality parameter^21^. Using terrain indices to model soil moisture conditions has successfully improved predictions of forest growth in numerous studies^25,51^.

The variation of site quality is not only driven by soil moisture conditions, but rather the effect of complex relationships between various environmental drivers. However, our study provides a unique insight into how soil moisture conditions drive site quality variation on a local landscape. To better understand the landscape-scale variation of site quality, the effects of additional biological and physical factors need to be studied. For example, soil physical and chemical properties have a large effect on site quality, however such information is challenging to extrapolate across a landscape scale.

## Conclusion

This study presents a landscape scale perspective on the relationship between forest site quality and soil moisture conditions within a managed boreal forest landscape. We estimated site quality across the entire study area using an age-independent difference height growth model based on repeated forest surveys and ALS scanning. Evaluation of site quality estimates showed lowest site quality in areas with the highest soil moisture levels. Although substantial variation was observed for estimated site quality, there was no distinct trend that was indicative of increased site quality in areas with intermediate soil moisture conditions. Collectively, our results deepen our understanding of how certain soil moisture conditions relates to growth potential across a heterogeneous boreal landscape.

### Supplementary Information

Supplementary Information.

## Data Availability

The dataset generated during the current study is available from the corresponding author on reasonable request.
