# Supplementary material for: Tree growth potential and its relationship with soil moisture conditions across a heterogeneous boreal forest landscape
Source: Sci Rep. 2024 May 9;14:10611. doi: 10.1038/s41598-024-61098-z (PMC11078985; doi:10.1038/s41598-024-61098-z)
Supplement: Supplementary file 1 — Supplementary Information. [file 41598_2024_61098_MOESM1_ESM.pdf]

Supplementary Information

S1 Spatial autocorrelation

The spatial autocorrelation of the estimated site quality was evaluated using a semivariogram; the results did not show any clear spatial autocorrelation.

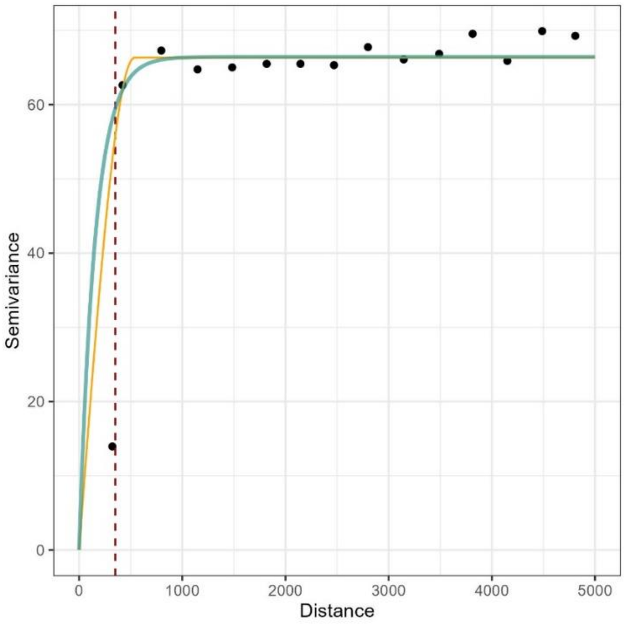

Figure S1. Semivariogram of the estimated site quality values, with exponential (blue) and spherical (yellow) functions fitted to the variogram.

S2 Dominant tree species

The effect of the dominant tree species on site quality was evaluated using a Kruskal-Wallis test (Kruskal-Wallis chi-squared = 13.089, df = 3, p-value = 0.004). When the Dunn-Bonferoni adjustment was performed, plots dominated by *Pinus contorta* were found to have significantly higher site quality than plots with other dominant tree species.

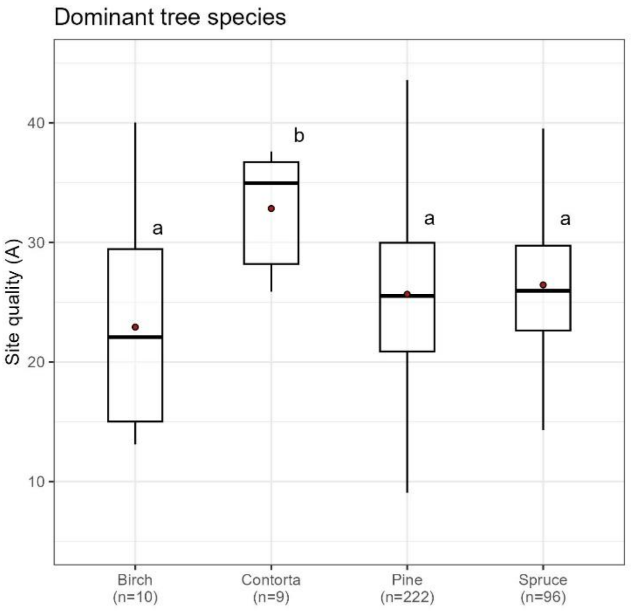

Figure S2. The relationship between dominant tree species and site quality, as calculated by a Kruskal Wallis test.
